# Frailty in Indigenous Populations: A Scoping Review

**DOI:** 10.3389/fpubh.2021.785460

**Published:** 2021-11-22

**Authors:** Ebony T. Lewis, Leanne Howard, Magnolia Cardona, Kylie Radford, Adrienne Withall, Adam Howie, Kenneth Rockwood, Ruth Peters

**Affiliations:** ^1^School of Population Health, Faculty of Medicine and Health, University of New South Wales, Kensington, NSW, Australia; ^2^School of Psychology, Faculty of Science, University of New South Wales, Kensington, NSW, Australia; ^3^Neuroscience Research Australia, Randwick, NSW, Australia; ^4^Gold Coast Hospital and Health Service, Southport, QLD, Australia; ^5^Institute for Evidence-Based Healthcare, Bond University, Robina, QLD, Australia; ^6^Geriatric Medicine Research Unit, Department of Medicine, Dalhousie University, Nova Scotia Health Authority, Halifax, NS, Canada

**Keywords:** frailty, indigenous, frail elderly, aging, prevalence

## Abstract

**Background:** Indigenous populations experience high rates of age-related illness when compared to their non-Indigenous counterparts. Frailty is a challenging expression of aging and an important public health priority. The purpose of this review was to map what the existing literature reports around frailty in Indigenous populations and to highlight the current gaps in frailty research within the Indigenous landscape.

**Method:** Scoping review of English language original research articles focusing on frailty within Indigenous adult populations in settler colonial countries (Australia, Canada, New Zealand and USA). Ten electronic databases and eight relevant institutional websites were searched from inception to October 2020.

**Results:** Nine articles met our inclusion criteria, finding this population having a higher prevalence of frailty and frailty occurring at younger ages when compared to their non-Indigenous counterparts, but two did not use a formal frailty tool. Females presented with higher levels of frailty. No culturally specific frailty tool was identified, and the included articles did not assess strategies or interventions to manage or prevent frailty in Indigenous peoples.

**Conclusions:** There was little definitive evidence of the true frailty prevalence, approaches to frailty screening and of potential points of intervention to manage or prevent the onset of frailty. Improvements in the quality of evidence are urgently needed, along with further research to determine the factors contributing to higher rates of frailty within Indigenous populations. Incorporation of Indigenous views of frailty, and instruments and programs that are led and designed by Indigenous communities, are crucial to address this public health priority.

## Introduction

The older population worldwide is growing at an unprecedented rate. In 2019, nine percent of the world's population was 65 years and over and this is projected to rise to 16% by 2050 (1). The increasing life expectancy is driven by public health developments and medical advancements (2). Older adults generate numerous societal benefits including contributing to paid and volunteer work, supporting loved ones and have the ability to pass experiences onto younger generations strengthening capacity (3). However, aging also presents challenges, as longer survival is associated with higher prevalence of chronic diseases, longer periods of demand for care and consequent burden on health and social support systems (1).

Patterns of aging amongst Indigenous People indicate faster growth than for non-Indigenous populations (2). Despite Indigenous populations having a relatively younger age structure (4), projections show that the number of older Indigenous Peoples is expected to significantly increase in the coming years. By 2051 the number of Aboriginal and Torres Strait Islander Australians aged 45 years and over is expected to increase from 167,170 in 2016 to 511,263 (5). Similarly, the number of First Nations seniors in Canada has doubled from 56,030 in 2006 to 121,665 in 2016 (6).

Indigenous Peoples worldwide tend to experience higher rates of age-related diseases than their non-Indigenous counterparts (4, 7). Overall, chronic diseases account for 70% of the health gap between Indigenous and non-Indigenous Australians (8); and frailty (9) associated with both aging and chronic illness has widespread implications for public health (10). These poorer health outcomes can be attributed to the long-term effects of colonization, intergenerational trauma and marginalization (11).

Frailty is a challenging expression of aging. It can be viewed as an individual's biological age rather than their chronological age (12) and has been characterized as a state of increased vulnerability to poor health outcomes as a result of age-related decline in reserve and function across multiple physiological systems (9, 13). A plethora of frailty tools exist in use within research and clinical practice (14) however, there is currently no consensus on an operational definition of frailty.

One widely used approach to frailty screening is the Fried Frailty Phenotype (FFP) (15), which assesses performance across five criteria including unintentional weight loss, self-reported exhaustion, physical inactivity, weakness (grip-strength) and slow walking. The presence of ≥ 3 criteria categorize a person as frail and one to two categorize an individual as pre-frail. A similar tool, the Edmonton Frail Scale (EFS) also assesses the frailty phenotype across nine components. Responses fall into five categories ranging from not frail to severely frail (16). The Frailty Index (FI) is another commonly used approach (12) defining frailty as accumulation of deficits, whereby a higher number of deficits the individual has indicates greater severity of frailty. This multidimensional approach includes deficits not only in the physical domain, but includes health conditions, cognition, disability, and psychosocial domains.

However, defined, frailty is consistently associated with poor outcomes including functional decline, aged-care admission, hospitalization and death (12, 17, 18). Both frailty and pre-frailty (the intermediate state between frail and robust) are prevalent among older adults in the general population. A systematic review of community-dwelling adults 65 years and over estimated frailty prevalence of 10.7% and pre-frailty 41.6% (19). It is important to note that given the lack of consensus on how to measure frailty and the varied diagnostic criteria, population prevalence can demonstrate significant variation making direct comparisons difficult.

Indigenous and non-Indigenous populations experience health differently. The World Health Organization defines health as a “state of complete physical, mental and social well-being and not merely the absence of disease or infirmity” (20). In contrast, Indigneous Peoples definition of health is more holistic and incoporates elements of physical, mental, spiritual and social well-being along with the broader health of family and the community as a whole; connection to land and culture are also important factors (21–23). This has important implications when characterizing and assessing frailty within Indigenous populations.

Given the potentially devastating impact of frailty and its associated adverse outcomes, there are implications for health service delivery, including screening and the development of prevention strategies. Therefore, to inform culturally appropriate strategies to prevent or delay the onset and progression of frailty it is important to estimate the prevalence of frailty within Indigenous populations and to establish whether the tools currently in use to measure frailty are culturally appropriate. As a first step to developing an understanding in this area we aimed to summarize the existing literature using standardized scoping review methodology.

The overall question for this scoping review was: What does the literature report on the extent of the problem, types of assessments and strategies to combat frailty within Indigenous populations?

Specifically, we aimed to map the (i) existing literature on the characteristics of frailty in Indigenous Peoples; (ii) tools that are being used to measure frailty and their characteristics; and (iii) strategies/interventions that have been developed to manage frailty in these populations.

## Methods

We searched MEDLINE, Prospero, Joanna Briggs Institute (JBI) and Open Science Framework and did not find any systematic reviews with a focus on frailty within Indigenous populations. Furthermore, our preliminary search found a paucity of research in this area. Therefore, we decided to conduct a scoping review as an appropriate way to map the existing literature (24). Scoping reviews are particularly relevant when the topic has not been extensively investigated, making it difficult to conduct a systematic review. This review sought to examine the range of research in this area, identify gaps in the literature, and provide insight into topics that may benefit from further evidence synthesis (25, 26).

This review followed scoping review methodology by Arskey and O'Malley (25) and advancements from the Levac et al. (24) framework and JBI (27). We did not exclude studies based on design and did not conduct quality appraisal.

Our review was registered in Open Science Framework on 8th October 2020 https://osf.io/u2f4p/.

### Inclusion Criteria

English language articles published or unpublished from inception to October 2020.

### Participants

This scoping review considered articles that included a focus on Indigenous adults 18 years and over from Western Nations. We chose to not limit our search to “older” adults as we wanted to keep our search as broad as possible. Articles were considered if >50% of participants were Indigenous, or results were reported separately for Indigenous participants. Articles were not excluded if participants had certain health conditions (e.g., diabetes).

### Concept

The main focus of this review was frailty, whether eligible articles included the concept as a primary or secondary goal. As there is no agreed upon definition of frailty, we considered articles where frailty measurement had been conducted using any frailty tools, instruments, measures, or scales. Given the anticipated dearth of literature around frailty in Indigenous populations, articles were also considered if participants have been identified by the author as frail without reporting on a frailty measurement, or where the measurement tool was not specifically designed for that purpose but has identified the partcipant as frail, in order to keep the review as broad as possible. Articles presenting findings from a frailty intervention, program, service or treatment administered to prevent, manage, or rehabilitate people at risk of or with established frailty were considered. The management of frailty (and/or prefrailty) is defined for the purpose of our study as any type of intervention or management strategy (diagnostic, therapeutic, evaluative) aimed to treat, slow down the progression of frailty, and/or reverse frailty levels. We considered method of measurement, accuracy or cultural appropriateness of instruments used in frailty prevalence estimates and assessment; and effectiveness of interventions measured by but not limited to: changes in overall frailty levels, improvement in frailty or general physical functioning and complications.

### Context

Articles that have explored frailty within the Indigenous landscape in any setting i.e., hospital, community-dwelling, residential aged-care and primary care in Australia, Canada, New Zealand and United States of America were included as Indigenous Peoples within these settler-colonies experience similar disadvantages and health disparities (28).

### Types of Articles

Published and gray literature meeting the inclusion criteria. Original research and all study designs were considered. Opinion pieces, editorials, letter to the editor, debate/discussion pieces, case studies and conference abstracts were excluded and documented with reasons. Systematic reviews were excluded, but their reference lists reviewed to identify additional articles. Although prior searches had not identified any systematic review that answered our research question, to ensure we captured the widest available evidence base, we reviewed the reference list of any related systematic review that were discovered in our searches. Given our limited resources, articles published in languages other than English were excluded.

### Search Strategy

We consulted and sought feedback on the search strategies from experts in the field of frailty and Indigenous health. We developed a preliminary search strategy and identified articles that reflected the aims of our review which were used to test the sensitivity of our search strategy. MESH, subject headings and keywords in abstract and title fields were used in ten databases: MEDLINE; EMBASE; PsycINFO; MEDLINE Epub ahead of print; CINAHL; Web of Science; Global health; AIATSIS Indigenous Studies Bibliography and ATSIhealth via informit; and Google Scholar. Specific terms differed slightly depending on the database, however, the main keywords were used throughout the search (Supplement 1). To ensure comprehensiveness of the search strategy, eight relevant institutional websites were also searched (Supplement 2).

### Article Selection

The lead reviewer carried out the database searches. Following the removal of duplicate records, references were imported into Covidence (29) a systematic review management software to support article selection.

Two reviewers (ETL,LH) independently screened all identified titles and abstracts and after reconcilliation of any discrepancies, full texts of agreed articles were obtained and independently read and assessed for relevance by the two reviewers. Any disagreements between the reviewers at each stage of the review (screening and full-text review) were solved by consensus or decision to include was made by a third reviewer (RP) who judged the eligibility of the article.

### Data Extraction

Data from each article was independently extracted from articles using a pre-defined purpose built data extraction form aligned with the research questions of this review (24) (Supplement 3). Consensus on reportable data items was reached through discussion, or was resolved with a third team member. The form was piloted on the first few articles. Charting of data was an iterative process, for instance additional unforeseen information can be charted (27). Therefore, refining the form at the review stage also occurred.

### Consultation

In line with Indigenous research principles and to ensure the prioritization of Indigenous voices and that findings are informed by Indigenous worldviews, this review was led by Indigenous researchers (ETL,LH,AW,AH) and there was ongoing consultation with Indigenous authors during every stage of the review.

## Results

**Figure 1:**
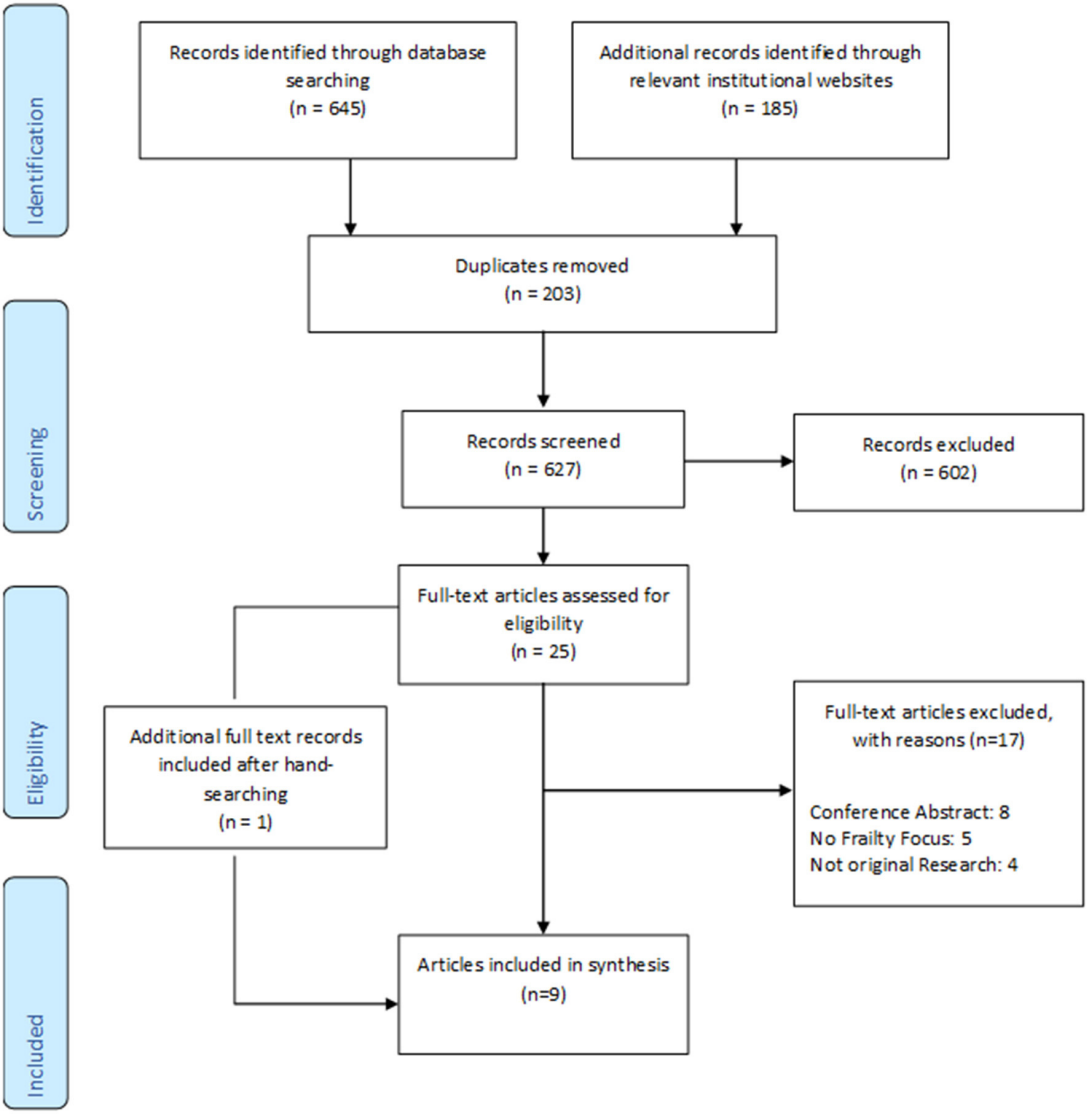
Flow Chart.

In total nine articles (30–38) met our inclusion criteria (Figure 1).

### Characteristics

The earliest eligible article was published in 1995 (38) with the remaining articles published from 2006 onwards. The number of Indigenous participants ranged in number from 22 to 1,820.

Three articles were Australian based (31, 32, 38), with a focus on Aboriginal Australians. Two articles (31, 32) included a sample population from the same cohort from Western Australia, with each article reporting on different outcomes. A further article (38) included an Aboriginal population in the Northern Territory.

Two articles were Canadian based (35, 37), reporting on First Nations Peoples. Both articles reported on data obtained from the same cohort included in the First Nations Regional Health Survey–Phase 2, with the earlier article (37) including a sample population of 250 First Nations communities in 10 participating regions of Canada, whereas the latter (35) reported on the Ontario cohort only.

Three articles were New Zealand based (30, 33, 34) with a focus on the Maori population and one article (36) investigating the American Indian population met our inclusion criteria.

Of the included articles, eight were in the community-dwelling setting, with one article conducted in hospitalized patients. Six articles were cross-sectional in design, two were longitudinal cohorts and one article was a needs assessment report.

Articles set in Australia, Canada and USA focused on Indigenous population only and the New Zealand articles incorporated both the Maori and non-Maori populations (30, 33, 34). Participants age ranged from 18–90+ years across the included articles. One article did not specify age range (38). Seven of the nine eligible articles provided gender participant information. In each of these articles, female participants were higher in number than male participants (Table 1).

**Table 1:** Study and population characteristics (*n* = 9 eligible articles).

**References**	**Country**	**Study Design**	**Total sample size (*N*=)**	**Sample size included in analysis (*N*=)**	**Sample size of Indigenous population (*N*=)**	**Study Period**	**Study Population**			
							**Inclusion criteria (participant characteristics)**	**age at baseline (years)**	**Gender at baseline % (*n*=)**	**Setting**
Hyde et al. (32)	Australia	Cross-sectional	289	141	141	2011–2013	Aboriginal Australians aged ≥ 45 years	62.2yrs–Mean age (range 45–88.9yrs)	F-68.1%	Remote−6 Aboriginal communities Kimberly region Western Australia
Hyde et al. (31)	Australia	Longitudinal cohort	363	363	363	2004–2013	Aboriginal Australians aged ≥ 45 years	60.7yrs	F-54.5%	Remote−6 Aboriginal communities Kimberly region Western Australia
Westerman (38)	Australia	A needs assessment	117	103 (includes children)83 (adults only)	83	1994–1995	Aboriginal Australians lacking independence in daily lives due to disability	-	F-57% (59)	Remote–East Arnhem District, Northern Territory
Slater et al. (35)	Canada	Cross-sectional	-	Sample size not reported, drawn on survey with weighted sample	Weighted sample 79,903	Aug 2008–Nov 2010	First Nations Canadians aged ≥ 18 years	Not specified (25–75+yrs) data presented	Not specified	24 First Nation communities across Ontario, Canada
Walker et al. (37)	Canada	Cross-sectional	1,820	1,820	1,820	1996–still underway	First Nations Canadians aged ≥ 18 years	-	F-54.2%	250 on-reserve and northern First Nation communities across Canada
Turner Goins et al. (36)	USA	Cross-sectional	411	411	411	July 2006–Aug 2008	American Indians aged ≥ 55 years	-	Not Specified	Southeast region of USA –community dwelling
Richards et al. (34)	New Zealand	Cross-sectional	420	420	22	-	Patients aged ≥ 18 years	Average age-68.2yrs Median age-73yrs	F-51.9% (218)	South Island (Christchurch & Burwood), New Zealand –Tertiary hospital
Kerse et al. (33)	New Zealand	Longitudinal cohort	937	937	421	2010–2015	Maori (≥80 years) and non-Maori (≥85 years)	-	F−58% (244)	Lakes District and Bay of Plenty Health Board areas (excluding Taupo area), New Zealand–community dwelling
Barrett et al. (30)	New Zealand	Cross-sectional	3,060	2,931	113	Feb 2000–April 2000	Aged ≥ 65 years	Singles-76yrs Couples-71yrs	F-74%	Community dwelling–permanent private dwelling New Zealand (72% North Island)

### Summary of Formal Frailty Measures

Of the included articles, seven incorporated the use of a formal frailty tool (Table 2). One article used the EFS, two articles used the FFP, and the remaining four articles used a FI. All frailty related data was reported by the authors as having been gathered from participants via questionnaires, interviews, or surveys. Four of the articles leveraged data collated from existing surveys (33, 35–37).

**Table 2:** A summary of formal frailty measures used in the included articles (*n* = 7).

**References**	**Country**	**Formal Frailty tool used**	**Frailty tool characteristics**	**Frailty cut-offs**	**Reason for frailty assessment**			**Comments on cultural adaptation/ appropriateness**
					**Risk**	**Outcome**	**Descriptive**	
Hyde et al. (32)	Australia	Frailty Index	28 items for FI	FI score ≥ 0.2 = Frail	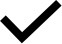	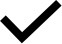	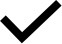	Participants administered a culturally specific questionnaire measures, including health conditions, Activities of Daily Living, and cognition
Hyde et al. (31)	Australia	Frailty Index	20 items for FI	FI score ≥ 0.2 = Frail	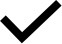		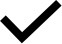	Culturally appropriate appropriate questionnaires were administered by research assistants to participants and family members / carers. Self-reported exhaustion, weight loss defined by person Body Mass Index
Slater et al. (35)	Canada	Frailty Index	26 items for FI	FI score ≥ 0.21 = Frail			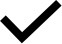	Data used to describe frailty among Ontario's First Nations adults from the First Nations Regional Health Survey-Phase 2 Ontario region, a First Nations–governed cross-sectional survey
Walker et al. (37)	Canada	Frailty Index	30 items for FI	FI score ≥ 0.2 = frailAdditional “pre-frail” category emerged for scores between 0.10 and 0.21.			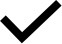	Data extracted from the First Nations Regional Health Survey-Phase 2–self reported. Based on the First Nations Regional Health Survey cultural framework
Turner Goins et al. (36)	USA	Fried Phenotype	5 components (weight loss, low energy expenditure exhaustion, weakness and slowness characteristics)	Robust = 0 components Pre-frail = 1 to 2 components Frail = 3 to 5 components			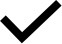	Data collected as part of the Native Elder Care Study. Information gathered through surveys administered by interviewers on disability, lower body functioning, personal assistance needs, physical and mental health conditions, psychosocial resources, and use of services
Richards et al. (34)	New Zealand	Edmonton Frail Scale	9 components (general heath, cognition, functional independence, self-reported health, social support, polypharmacy, mood, continence and functional performance)	Score of 8 or more considered frail Mild frailty = score 8 to 9 Moderate frailty = score 10 to 11 Severe frailty = score 12 to 18 Apparently vulnerable = score 6 to 7			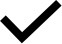	Trained clinicians were assigned in pairs to different wards. Frailty assessment questions were referenced to the time of admission
Kerse et al. (33)	New Zealand	Fried Phenotype	5 components (weight loss, low energy expenditure exhaustion, weakness and slowness characteristics)	3 of 5 key measures-higher scores indicate greater frailty	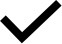	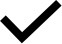	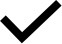	Data gathered from the LiLACS NZ study. All participants undertook a short core questionnaire with most completing a longer questionnaire by interview

#### Frailty Index

The four articles that used a FI (31, 32, 35, 37) calculated the FI using between 20 and 30 deficits. Deficits included Activities of Daily Living, health conditions, cognition and mood. Across all four articles reporting on the FI, a participant was considered frail if the score was ≥0.2 with one article (37) also including a pre-frailty score between 0.10 and 0.21. Two articles (31, 32) reported that they had included the use of a tailored culturally specific questionnaire that was administered by research assistants to participants, families and/or caregivers.

#### Fried Frailty Phenotype

Two articles (33, 36) used the FFP and participants were classified as frail if scoring ≥3 across the five criteria of weight loss, energy, exhaustion, slowness & weakness. In addition, Turner Goins et al. (36) classified participants as pre-frail if scoring 1–2 criteria. Data was gathered via interviewer-administered questionnaires.

#### Edmonton Frail Scale

One article employed the use of the EFS (34). Participants were classified as frail if scoring ≥ 8 across nine health, social, and functional components. Data was gathered via frailty assessment questions administered by trained clinicians.

Three articles assessed frailty as an assessment of risk i.e., where frailty can predict poor outcomes, two articles included frailty as an outcome measure and in seven articles frailty was used as a descriptive measure, for example reporting on the prevalence.

### Prevalence of Frailty

Eight articles reported the prevalence of frailty amongst Aboriginal Australians, First Nations Canadians, New Zealand Maori and an American Indian population (Table 3). For longitudinal studies we have reported the prevalence at baseline.

**Table 3:** Articles that included frailty prevalence in articles conducted among Indigenous Peoples (*n* = 8).

**References**	**Country**	**Indigenous Population**	**Location**	**Setting**	**Rurality**	**Age**	**Year/s of data collection**	**Frailty tool**	**Indigenous Frailty prevalence at baseline (%)**					**Differences between Indigenous and non-Indigenous frailty prevalence**
									**Robust**	**Pre-Frail**	**Frail**	**Male**	**Female**	
Hyde et al. (32)	Australia	Aboriginal Australians	Kimberly Region, Western Australia	Community-dwelling	Remote	45+ years	2011–2013	Frailty Index	-	-	59.6%	-	-	-
Hyde et al. (31)	Australia	Aboriginal Australians	Kimberly Region, Western Australia	Community-dwelling	Remote	45+ years	2004–2006	Frailty Index	-	-	Overall 65.3% (95% CI 60.1–70.2) 45–49 yrs–54.9% (95% CI 42.7–66.8) 80+ yrs–83.3% (95% CI 65.3–94.9)	-	-	-
Westerman (38)	Australia	Aboriginal Australians	East Arnhem District, Northern Territory	Community-dwelling residing in East Arnhem District	Remote	All ages (49% >55 years)	1994–1995	No formal frailty tool used	-	-	26%	43%	57%	-
Slater et al. (35)	Canada	First Nations Canadians	Communities across Ontario	Community-dwelling	-	25+ years	2008–2010	Frailty Index	-	-	25–34 yrs−7.3% 35–44 yrs–14.8% 45–54 yrs–26.1% 55–64 yrs–41.9% 65–74 yrs–50.1% 75+yrs-47.4%	21.3%	26.0%	65–74 yrs and 75+yrs in First Nations 50.1%, 47.4% and 16.0%, 33.5% of non-Indigenous respectively
Walker et al. (37)	Canada	First Nations Canadians	On-reserve and northern First Nation communities	On-reserve and community-dwelling	Rural and remote - 51%	18+ years	1996–2010	Frailty Index	-	-	65+yrs-47.3% (95% CI 43.9–50.6)	40.7% (95% CI 36.1–45.4)	52.8% (95% CI 48.5–57.1)	-
											35–64 yrs	17.8% (95% CI 16.0–19.7).	23.1% (95% CI 21.0–25.4)	
Turner Goins et al. (36)	USA	American Indians	Southeast region of USA	Community-Dwelling	-	55+ years	2006–2008	Fried Phenotype	52.8%	44.3%	2.9%	-	-	-
Richards et al. (34)	New Zealand	Maori	South Island (Christchurch and Burwood)	Tertiary Hospital		18+ years	-	Edmonton Frail Scale	36.4%		63.6%	-	-	Maori had higher frailty in comparison to non-Maori OR-4.00, 95% CI 1.45–11.90, *p* = 0.01
Barrett et al. (30)	New Zealand	Maori	72% of respondents lived in the North Island	Community dwelling -permanent private dwellings	67%-urban areas 24%-minor urban areas 9%-rural areas	65+ years	2000	-	-	-	11.5%	7%	8.9%	Maori-11.5% Non-Maori-7.9%

Although the population and age groups are not directly comparable, frailty prevalence estimates from three Australian studies of remote community-dwelling Aboriginal adults suggest that using a formal frailty tool and targeting older adults (31, 32) yields a much higher frailty prevalence than not using a formal frailty tool (38) among all adults 18 years and over. This finding is also true for the New Zealand studies (30, 34), even when using a standard tool across all age groups 18 years and over.

The Canadian studies using the FI in community-dwelling First Nations adults in two different years consistently found that half of the older participants (65+yrs) were categorized as frail. The most recent study (35) also reported much lower frailty prevalence for younger age groups.

The recent study of younger (55+yrs) American Indians using the FFP yielded a very low prevalence of frailty and a sizeable pre-frailty estimate (36).

The only study assessing Indigenous patients in a hospital setting (34) reported one of the highest frailty prevalence rates for adults 18 years and over, only exceeded by the eldest (80+yrs) subgroup from community-dwelling Aboriginal Australians (31).

Only three studies reported frailty prevalence difference between the Indigenous and non-Indigenous population (30, 34, 35), and generally found higher frailty prevalence among Indigenous adults. However, one study (30) did not report statistical significance.

### Key Findings and Association of Frailty and Poor Health Outcomes

Several studies also reported on the associations between Indigenous frailty and poor outcomes. The most notable were a trend of early onset of frailty among Maori and First Nations Canadians compared to their non-Indigenous counterparts; association between frailty and mortality in Australia; correlation between dementia and frailty in New Zealand; and positive correlation between poor glucose control and higher prevalence of frailty (Supplement 4).

### Potential Sources of Bias

Although we did not do a formal risk of a bias assessment as this is a scoping review, it is important to note that the included articles have potential sources of bias. For example small sample size (31, 32, 34, 37, 38), non-response bias (31, 32), false positives and negatives i.e., risk of misclassification of frailty within the earlier articles which did not utilize a formal frailty tool (30, 38) and a lack of representative populations where frail individuals may decline to participate in research (36) (Supplement 5).

### Interventions

None of the eligible articles included any interventional approaches or strategies to the management or prevention of frailty or those at risk of frailty amongst Indigenous populations.

## Discussion

This scoping review has highlighted the scarce evidence available regarding the understanding, impact and measurement of frailty within Indigenous populations in the context of settler-colonies. After an extensive literature search, nine articles met the inclusion criteria. Despite efforts made to include Indigenous perspectives of frailty (Table 2), this limited evidence highlights multiple shortcomings including inconsistent approach to measurement; not using standard instruments to estimate frailty prevalence; common absence of comparison of frailty results with mainstream population groups; and lack of monitoring of frailty prevalence over time. Across the included articles, there is no consensus pertaining to target age group for estimation of frailty. In addition, the lack of standardization of a frailty measurement tool and absence of commentary on appropriateness for Indigenous populations limits the validity of the estimates and comparison between articles. Several frailty definitions were used and therefore the true Indigenous frailty prevalence was difficult to determine and compare across Indigenous population groups (9). There was also a vast difference in frailty prevalence both within countries and globally. This may be a consequence of not only differences in populations, but varying frailty definitions and inclusion of different age groups and underlying comorbidities.

What we could determine from the limited evidence available is most studies found Indigenous Peoples to have higher rates of frailty and younger onset compared to their non-Indigenous counterparts. We also found Indigenous women were more likely to experience frailty compared to Indigenous men. This is consistent with findings from non-Indigenous populations (39). Furthermore, the Indigenous women sample size included in the articles was larger than the male cohort, which could potentially have skewed the results.

Two of the earliest included articles did not report on the use of a formal frailty tool. This is not surprising as frailty is a relatively new concept within the literature but detracts from the validity of results. The remaining seven articles that used a formal frailty tool were all published between 2016 and 2020. One-time prevalence of frailty could be informative when there is no prior source of data, but time-trends would have more utility to plan healthcare and community resources to cater for the changing needs of individuals and of the population.

Our search did not find any interventional articles to manage or address frailty within Indigenous populations or those at risk of frailty. Interventions such as strength and resistance training and nutritional supplements have been found to prevent or delay frailty onset (40). Future studies could investigate the feasibility, acceptability and effectiveness of these approaches in Indigenous populations to fill this important knowledge gap.

Context also play an important role in gaining true insights into prevalence. All included articles were in community-dwelling settings, apart from one based in hospitalized patients (34). Hospitalized patients are more likely to be in poorer health and experiencing higher rates of frailty. Whereas, community-dwelling individuals may have lower levels of frailty as they are in better health and able to manage independently in their daily lives (36). Others have found high frailty prevalence in hospitalized patients (41).

A number of the included articles highlight the correlation between frailty and other chronic diseases (32, 33). Chronic diseases are responsible for almost two-thirds of the estimated burden of disease within Indigenous Australians (8). Furthermore, the prevalence of multimorbidity in Indigenous Peoples was found to be nearly 2.6 times that of non-Indigenous population and the adjusted hazard of mortality within twelve months was 2.4 times as high, and 1.5 times that of the non-Indigenous population after adjusting for the number of morbidities (42). Whilst chronic diseases were found to contribute to a higher prevalence of frailty, highlighting the importance of screening for frailty in routine care. The reported associations between dementia, diabetes, and frailty or that of frailty and mortality found in our review cannot be used to infer causality due to the cross-sectional nature of these studies.

Frailty may present earlier within Indigenous Peoples due to overall poorer health and social outcomes across the life-span (43) and contribute to the lower life expectancy amongst Indigenous populations. This is consistent with findings related to other age-related conditions such as dementia (7), and should be an incentive to identify and evaluate programs with social and health components to prevent or reduce frailty and its early onset.

After reviewing all articles, it was evident that Indigenous perspectives had not been considered when defining frailty or incorporated into the design of the discussed frailty measurement tools. The included definitions and tools are based on Western models and largely adopt a deficit-based approach. While some included studies appeared to have attempted some level of cultural adaptation and training of people to administer surveys, and a study reporting the use of a culturally appropriate questionnaire (31). Deficit-based understandings of health are inconsistent with strength-based Indigenous models of well-being. Current deficit-based approaches are key limitations in assessing frailty, as aging becomes framed in a negative light. In addition, there is a need to highlight the importance of strength and resilience of Indigenous Peoples (44) along with promoting culturally safe and supportive healthcare. Instruments with face validity and culturally appropriate domains need to be developed.

Holistic approaches to defining and assessing frailty are required for this to be closely aligned with Indigenous definitions of health and perspectives on aging, incorporating social, emotional and cultural well-being of the community as a whole (21). Social and cultural factors that reflect the experiences of Indigenous Peoples including early life experiences, social capital, cultural connectivity, language, and engagement in traditional culture (45, 46) could also be considered in future research investigating frailty in Indigenous populations. Frailty tools could also be strengthened with assessment items including characteristics such as resilience, vitality, individual and community strengths as opposed to the current deficit frameworks. A standardized setting-specific measurement framework and approach across Indigenous populations in various locations or services would help inform a more accurate understanding of the true frailty prevalence within this population. Indigenous Peoples are well positioned to drive these developments (47), drawing upon Indigenous knowledge, experiences and ways of knowing.

Addressing modifiable lifestyle risk factors across the life-course may help lower the risk of frailty, delaying or preventing onset (48). Culturally appropriate programs across the lifespan, including targeting age-related conditions in earlier life may have the potential to reduce the prevalence of early onset of frailty (13). Further research is essential, reflecting Indigenous knowledge to help with firstly understanding the current environment and potentially lead to informing culturally appropriate strategies. Indigenous views and understanding of frailty as well as co-designed approaches to screening is urgently needed.

Socioeconomic circumstances and frailty are heavily intertwined. Addressing the underlying social and cultural determinants (46), which result in the differences experienced between Indigenous and non-Indigenous populations, should be investigated. It is worth examining whether factors such as higher educational attainment and improved employment opportunities through the life-course may lower frailty risk in later life.

Overall, a sophisticated multi-sector focus is required to tackle the determinants of frailty, strongly underpinned by research and collaboration with Indigenous communities. This would create a strong foundation to plan and better care for the aging Indigenous population.

### Limitations

An important limitation to this review is the heterogeneity between approaches to frailty assessments and the heterogeneity within Indigenous populations. Importantly, given the diversity of Indigenous Peoples and that currently, there is no universally agreed definition of Indigenous Peoples, the fundamental rights-based criterion in Indigenous identification is self-identification (49). For this review, we sought to capture the widest possible evidence base relevant to Indigenous populations, broadly and inclusively defined. Our review captured articles that explicitly used the term “frailty,” so it is possible that relevant articles that may have measured aspects of frailty but did not describe it as such may have been missed. In addition, although a rigorous search strategy was employed including both published literature and relevant institutional websites, articles may have been missed. It is also important to note that this search was confined to Indigenous Peoples within settler-colonies, therefore these results cannot be generalized to other Indigenous populations.

While evidence suggests the age structure of Indigenous populations is changing faster than that of many non-Indigenous populations, there remains a paucity of research on the underlying drivers of Indigenous aging. Nevertheless, it is important for us to understand the culturally relevant attributes amongst aging Indigenous populations.

## Conclusion

To our knowledge this is the first review to map what the literature reports around frailty within Indigenous populations. Despite the large health burden frailty seemingly has on Indigenous Peoples, there was little definitive evidence of the true frailty prevalence, approaches to frailty screening and potential points of intervention to manage or prevent the onset of frailty amongst Indigenous Peoples. Improvements are urgently needed in the quality of evidence around frailty and its apparent earlier onset amongst this population. Furthermore, this review highlights an urgent need for further research to determine the factors contributing to these higher rates of frailty within Indigenous populations and recommends that Indigenous views of frailty must be incorporated into existing research as co-designers of frailty instruments and strategies and interventions to address this important public health priority.

## Data Availability Statement

The original contributions presented in the study are included in the article/Supplementary Material, further inquiries can be directed to the corresponding authors.

## Author Contributions

ETL conceived the idea. ETL and LH screened the articles and led the initial draft and all authors made critical revisions to the manuscript. All authors participated in the discussions that led to the design of this review and contributed substantially to the development of this work and reviewed, and provided final approval of the version to publish.

## Funding

ETL was supported by a UNSW Scientia PhD Scholarship.

## Conflict of Interest

The authors declare that the research was conducted in the absence of any commercial or financial relationships that could be construed as a potential conflict of interest.

## Publisher's Note

All claims expressed in this article are solely those of the authors and do not necessarily represent those of their affiliated organizations, or those of the publisher, the editors and the reviewers. Any product that may be evaluated in this article, or claim that may be made by its manufacturer, is not guaranteed or endorsed by the publisher.
