# Supplementary material for: Frailty in Indigenous Populations: A Scoping Review
Source: Front Public Health. 2021 Nov 22;9:785460. doi: 10.3389/fpubh.2021.785460 (PMC8646043; doi:10.3389/fpubh.2021.785460)
Supplement: Supplementary file 1 [file Table_1.DOCX]

Supplementary Material

**Supplement 1: Search Strategy**

| **Example search strategy MEDLINE** |
| --- |
| Indigenous.ab,ti OR Indigenous Peoples/ OR Aborigin*.ab,ti OR Torres Strait Island*.ab,ti. OR Oceanic Ancestry Group/ OR Inuits/ OR Maori.ab,ti OR American Indian*.ab,ti OR Indians, North American/ OR Native American*.ab,ti OR First Nation*.ab,ti OR First People*.ab,ti OR Metis.ab,ti OR Tribe*.ab,ti OR Alaska Native*.ab,ti OR Alaska natives/ OR Inuit*.ab,ti OR Native Hawaii*.ab,ti ORIwi.ab,ti OR Eskimo*.ab,ti OR Tribal*.ab,ti OR Koori*.ab,ti OR Murri*.ab,ti.  AND  Frailty/ OR Frail Elderly/ OR Frail*.ab,ti OR prefrail*.ab,ti OR pre-frail*.ab,ti OR nonfrail*.ab,ti OR non-frail*.ab,ti OR Fragility.ab,ti OR Debility.ab,ti OR Deconditioning.ab,ti OR Oligophreni*.ab,ti |

**Databases searched**

- MEDLINE
- EMBASE
- PsycINFO
- MEDLINE Epub ahead of print and in-process & other non-indexed citations
- CINAHL
- Web of Science
- Global health
- AIATSIS Indigenous Studies Bibliography via informit
- ATSIhealth via informit
- Google Scholar

In Google Scholar, we used the first 100 references sorted by relevance, as the number of references from other databases was low and therefore we expected to reach saturation of potentially relevant references by this number (Bramer et al., 2017). We also logged out of Google Scholar to prevent our search results being customised based on previous search history. All searches were conducted in October 2020.

**Websites searched**

- Australian Indigenous HealthInfoNet
- New Zealand Ministry of Health
- Lowitja Institute
- Health Canada
- National Collaborating Centre for Indigenous Health
- Canadian Frailty Network
- World Health Organization
- US Department of Health & Human Services Office of Minority Health

Reference

Bramer WM, Rethlefsen ML, Kleijnen J, Franco OH. Optimal database combinations for literature searches in systematic reviews: a prospective exploratory study. Systematic reviews. 2017;6(1):245.

**Supplement 2: Recordkeeping and approach to website searches**

Websites were searched by adapting Stansfield and colleagues (Stansfield et al., 2016) three staged approach of:

1) Planning search – identifying websites

2) Executing search – identifying keywords

3) Screening records and managing results

Keywords were entered into the search or advance search fields (where available) of the website. Where the option was available, title and abstract were searched. All resulting articles were documented in an excel spreadsheet which included website name, keywords used in the search, search date, author, date of publication, title and URL. Where a website yielded search results of ≥100 articles, the first 100 articles were included as saturation was assumed to have been reached at that point. Title screening was conducted on identified articles, with potentially eligible articles followed up with full text review. Final reviewer decision on eligibility of article for inclusion was recorded.

| **Name of website** | **Number of promising articles for consideration** | **Number Scanned** | **Approach to screening, e.g., title, then abstract/full text OR first 100 ranked by relevance** | **Notes** |
| --- | --- | --- | --- | --- |
| Australian Indigenous Health*Info*Net  <https://healthinfonet.ecu.edu.au/> | 4 | 7 | Title then abstract/full text | Key words frailty, frail |
| Lowitja Institute  <https://www.lowitja.org.au/> |  | 0 |  | Search did not yield and results (key words used frail, frailty) |
| National Collaborating Centre for Indigenous Health  <https://www.nccih.ca/en/> |  | 0 |  | Search did not yield and results (key words used frail, frailty) |
| World Health Organisation  <https://www.who.int/> | 0 | 26 | Title then abstract/full text | Key words Aboriginal Frail/Frailty, Indigenous Frail/Frailty |
| Ministry of Health NZ  <https://www.health.govt.nz/our-work/populations/maori-health> | 1 | 34 | Title then abstract/full text | Key words Maori Frail/Frailty, Indigenous Frail/Frailty  First Nation Frail/Frailty |
| Health Canada  <https://www.canada.ca/en/health-canada.html> | 6 | 100 | First 100 by each search term ranked by relevance | Key words “Indigenous” “Aboriginal” “Inuit” “First Nation” and “Frail*/frail” |
| Canadian Frailty Network  <https://www.cfn-nce.ca/> | 3 | 7 | Title then abstract/full text | Key words Indigenous, Inuit, First Nation, Aboriginal |
| US Department of Health & Human Services Office of Minority Health  <https://www.minorityhealth.hhs.gov/> | 1 | 11 | Title then abstract/full text | Key words frailty, frail, American Indian |

Reference

Stansfield C, Dickson K, Bangpan M. Exploring issues in the conduct of website searching and other online sources for systematic reviews: how can we be systematic? Systematic reviews. 2016;5(1):191.

**Supplement 3: Data Extraction**

| Reviewer Name |  |
| --- | --- |
| Article title (short title if necessary) |  |
| Author(s) (if >3 only record the first three) |  |
| Year of publication |  |
| Country/location (where the source was published and/or study conducted) |  |
| Study population  -age of participants  -mean age of participants  - gender  -setting  -Indigenous specific population  -location (if known)  -rurality (remote/urban) |  |
| Research purpose/aims |  |
| Study design |  |
| Total sample size |  |
| Sample Size - included in the analysis |  |
| Frailty definition specified |  |
| Frailty tool (measure, scale) used in the study |  |
| Frailty tool characteristics (if specified) |  |
| Frailty cut-offs |  |
| Non-frailty tools (measures, scales) |  |
| Ad hoc measures |  |
| Frailty prevalence:  -robust  -prefrail  -frail |  |
| Frailty incidence |  |
| Purpose of frailty measurement |  |
| Adverse outcomes associated/ predicted with frailty |  |
| Interventions |  |
| Description of any intervention, management strategies or services targeting frailty (if applicable) |  |
| Other key findings of relevance to the review questions and/or other outcome(s) |  |

**Supplement 4: Key findings and association of frailty with poor outcomes**

| **Authors and publication year** | **Country** | **Indigenous Population** | **Research Purpose/Aims** | **Key Findings** |
| --- | --- | --- | --- | --- |
| **Hyde et al., 2019** | Australia | Aboriginal Australians | To determine If HbA1c levels (glycemic controls) were associated with frailty | Trend for greater frailty experienced by higher category of HbA1c p=0.025  Participants with a low body mass index and obesity had higher FI score than those of normal body weight, association between frailty and obesity may reflect an overweight phenotype where muscle wasting and excess fat co-exist – high frailty prevalence in obese participants |
| **Hyde et al., 2016** | Australia | Aboriginal Australians | Determine incidence and incidence of frailty and associations between frailty, disability and mortality | Frailty at baseline strong predictor of all-cause mortality (multivariate W1 HR=2.6 95%CI 1.2-3.0) No significant association with disability  20 item FI used however by W2 a larger 28 item FI was used, authors compared prevalence at wave 2 using 2 indexes and found no statistically significant difference in prevalence  54.9% of 45–49yrs remote Aboriginal people already classified as frail and frailty rates continued to increase significantly in older age categories, up to 83.3% in aged 80 and older |
| **Westerman 1995** | Australia | Aboriginal Australians | Identify number of people with handicaps and identify major problems & areas of need | The most pressing needs for Aboriginal carers in the East Arnhem District was respite  Of the 103 handicapped/frail aged people residing in East Arnhem District there are 26 people with handicaps whose carers require respite now - 25% (26/103)  26% of the handicapped participants were also frail aged |
| **Slater et al., 2020** | Canada | First Nations Canadians | Provide an Ontario wide profile on ageing in First Nations & relationship between frailty, age & sex | Females higher proportion of frail 26.0% vs men 21.3% - however no difference by age group  First Nations adults experienced higher frailty levels than the overall Canadian population  Similar to other chronic diseases, early onset frailty appears to affect First Nations Communities  First Nations People residing in First Nations communities experienced more frailty at younger ages |
| **Walker et al., 2017** | Canada | First Nations Canadians | Frailty index validated in the general older population to be applied to describe ageing experiences in First Nations populations residing on-reserve and in northern communities. | First Nations frailty levels in 45–54yrs were similar to those aged 65–74 years in the general population, has consequences for supportive services requirements |
| **Goins et al., 2019** | USA | American Indians | Estimate prevalence of frailty and identify its correlates | Adjusted analysis found association between pre-frail and frail classification with younger age  Lower education, additional chronic conditions, increased ADL limitations had greater likelihood of pre-frailty or frailty |
| **Richards et al., 2019** | New Zealand | Maori | To evaluate the prevalence of frailty amongst adult inpatients in a tertiary hospital | Frailty prevalence increased with age; patients aged 85+yrs were more likely to be frail than those aged under 65  Maori patients had greater frailty compared to NZ European population (OR = 4.00, 95% CI 1.45–11.90, p = 0.02). Higher prevalence amongst Maori at a younger age  Patients admitted for medical or surgical specialty less likely to be frail  Patients admitted for rehabilitation more likely to be frail  Frail patients more likely to require a rest home or hospital level care  Frailty was higher in female patients with 117 of 218 (53.9%) classified as frail compared to 88 of 202 (43.6%) of male patients |
| **Kerse et al., University of Auckland, 2017** | New Zealand | Maori | To determine predictors of successful advanced ageing and understand trajectories of  health and wellbeing  To highlight several common health conditions of advanced age and examine the impact on frailty, functional status, quality of life and health service use when dementia symptoms also present | Dementia associated with higher frailty with Maori experiencing higher prevalence of dementia than non-Maori  No notable difference in dementia/cardiovascular disease (CVD) related differential for frailty scores between non-Maori and Maori, females and males, or by socioeconomic characteristics  Dementia increased association of diabetes mellitus (DM) with frailty  Participants with dementia alone had worse frailty than those with DM or CLD alone. Frailty was higher for those with CVD alone  Maori less likely to finish school and more likely to smoke leading to CVD increasing risk of frailty |
| **Barrett et al., 2006** | New Zealand | Maori | Begin to develop a descriptive account of characteristics of older people who live in the community and become frail, as a way of informing preventive care programs and measures to support functional needs | Frailty prevalence among Maori in 65–70yrs was the same as non-Maori in the 81–84yrs, suggesting a 10 to 15yr difference in frailty onset within the Maori population. Greater than the 8yrs difference in life expectancy between non-Maori and Maori for the year 2000  Peak of frailty within the Maori participants was in the 75–80yrs and likely reflects the accumulation of health problems and losses of function associated with nearing end of life  Different history of opportunity likely to contribute to higher rate of frailty amongst Maori at a younger. Disparities between non-Maori and Maori over the lifespan can have a cumulative effect manifesting in later years as frailty |

**Supplement 5: Potential bias and limitations of articles**

| **Authors and publication year** | **Country** | **Possible Bias / Limitations** | |
| --- | --- | --- | --- |
| **Hyde et al., 2019** | Australia | Small sample size due to remoteness and cost of field work  Recall and response bias of participants – trained Aboriginal research assistants were used to mitigate this  Non-response bias, particularly as wave 2 – possible prevalence may have been overestimated  Frailty index comprised only 28 items, less than the minimum 30 suggested by Searle et al.(Searle et al., 2008) |  |
| **Hyde et al., 2016** | Australia | Small sample size due to remoteness and cost of field work  Recall and response bias of participants – trained Aboriginal research assistants were used to mitigate this  Non-response bias, particularly as wave 2 – possible prevalence may have been overestimated  Frailty index comprised only 20 items, less than the minimum 30 suggested by Searle et al.(Searle et al., 2008) |  |
| **Westerman 1995** | Australia | Small sample size  Sample population are all handicapped  Almost half of the participants have a severe disability (47)  Frailty tool not used  Older article - conducted 25yrs ago, improvements in frailty measurement tools since this time |  |
| **Slater et al., 2020** | Canada | No inclusion of Indigenous perspective of frailty |  |
| **Walker et al., 2017** | Canada | Sample population only includes older people remaining healthy enough to continue living in First Nations communities, possible survivor bias  Which older people continue residing in First Nations communities is determined by health policy  Frailty possibly underestimated due to limited data on the important age-related conditions of dementia and urinary incontinence  Standardising age and sex required for a true population comparison  Deficit based approach |  |
| **Goins et al., 2019** | USA | Findings indicated a relatively low prevalence of frailty. Reasons could include:  Frail individuals more likely to decline participation in study  Participants had a high prevalence of obesity (56%)  Contribution of BMI to muscle mass in American Indians outweighing its contribution to the percentage of fat mass |  |
| **Kerse et al., University of Auckland, 2017** | New Zealand | Focus of article is dementia, relationship with frailty is a secondary consideration |  |
| **Richards et al., 2019** | New Zealand | Small sample size  Data derived from a single institution over one day, relatively homogeneous ethnicity, may not result in similar findings in other centres |  |
| **Barrett et al., 2006** | New Zealand | Older article - conducted 14yrs ago, improvements in frailty measurement tools since this time  Formal frailty tool not used  Likely to include false negatives and false positives in subgroup defined as frail due to selection criteria  Fluctuating nature of frailty is not captured in  Prevalence of frailty lower among home owners, however those experiencing frailty most likely sell the family home to move into supported accommodation |  |
